# Determining risk factors of continuous renal replacement treatment after emergency surgery for type A acute aortic dissection: statistical issues

**DOI:** 10.1186/s13019-021-01396-z

**Published:** 2021-03-29

**Authors:** Zhao-Jing Xue, Peng Dong, Fu-Shan Xue

**Affiliations:** Department of Anesthesiology, Beijing Friendship Hospital, Capital Medical University, NO. 95 Yong-An Road, Xi-Cheng District Beijing, 100050 People’s Republic of China

**Keywords:** Severe acute kidney injury, Continuous renal replacement treatment, Risk factors, Long-term outcomes

## Abstract

This letter to the editor has made several comments regarding possible statistical issues in recent article by *Wang* et al. determining the risk factors of continuous renal replacement treatment after emergency surgery for type A acute aortic dissection, which is published in *Journal of Cardiothoracic Surgery.* 2020; 15(1):100. Our comments were involved in the issues of using the propensity score matched cohorts to adjust the covariates that can potentially confound the primary outcomes, process of establishing multivariate model and application of Kaplan-Meier curve analysis in this retrospective study. We would like to remind readers to pay special attention to these issues and invite the authors to comment on these.

**To the Editor:**

With great interest, we read the recent article by Wang et al. [1] determining the independent risk factors and long-term outcomes for postoperative continuous renal replacement treatment (CRRT) in patients undergoing emergency surgery for type A acute aortic dissection. By the binary logistic regression analysis and propensity score matching, they showed that preoperative serum creatinine and cardiopulmonary bypass (CPB) time were the independent risk factors for postoperative CRRT. Other than the limitations described by the authors in discussion section, however, there were other statistical issues in this article that we would like to remind readers for attention and invite the authors to comment on these.

First, this study applied the propensity score matching to adjust the potential influences of baseline characteristics (demographic variables, previous medical history, aortic dissection features and pericardial effusion) on the primary outcomes. However, a significant limitation of the propensity score matching is that the patients who cannot be matched are excluded from data analysis. As the excluded patients from data analysis often are the preoperative sickest and the healthiest patients, only the patients with moderate co-morbidities are remained for statistical comparison between the two propensity-score matched cohorts [2]. For example, before the propensity score matching in this study, the patients without postoperative CRRT were younger, had less comorbidities (hypertension, coronary artery disease and pericardial effusion), and lower preoperative serum creatinine and blood urea nitrogen levels. To generate two matched propensity-score cohorts with relatively small imbalances in given covariates, the authors performed one-to-one pair matching using nearest neighbor matching without replacement within 0.02 standard deviations of the logit of the propensity score as caliper width. After the propensity score matching, 95.2% of control patients without postoperative CRRT and 74.9% of patients with postoperative CRRT were excluded from data analysis. As many of included patients were excluded from data analysis, we are concerned that the comparative results of two propensity-score matched cohorts may bias from the findings from the real-world clinical population of patients. Most important, a major need of the propensity score matching is that all known factors affecting the primary outcome should be taken into the account. Evidently, it is not true in this study. The available evidence indicates that preoperative cardiogenic shock and ventilator use, sepsis, malperfusion complications, intraoperative large blood transfusion and prolonged CPB time are associated with an increased risk of severe or persistence acute kidney injury requiring CRRT after surgery for type A acute aortic dissection [3–5]. This limitation can further reduce the accuracy and inferences of the propensity-score matched cohorts for adjustment of potential confounders.

Second, in the statistical analysis section, the authors described that the multivariate model included variables that were significant on the univariate analysis. However, the readers were not provided the results of univariate analysis. According to the data in Table 3 of this article, the included variables in the multivariate model were those that were significant in the initial comparisons of demographics and perioperative variables between patients with and without CRRT, rather than real results of the univariate analyses for demographics and perioperative data. As a general principle of establishing multivariate model, after the initial dataset analysis of two matched cohorts, the variables significantly with statistical differences between patients with and without CRRT, defined as *P* < 0.05, should are further incorporated into the univariate analysis to examine the multicollinearity among candidate independent variables. Then, the variables with large *P* values (*P* < 0.2) in the univariate analysis are included into the multivariate model using CRRT as the dependent outcome variable to identify the risk factors of CRRT. By such adjustment of patients’ baseline characteristics and controlling of selection biases, the independent risk factors for CRRT can be identified [6]. Based on the odds ratio, 95% confidence interval and *P* values of the risk factors obtained by the multivariate analysis, the reader can determine the independent contribution of each risk factor to the need of CRRT. We argue that clarifying above issues of the multivariate modeling would improve the transparency and interpretation of findings from this study.

Finally, in this study, the Kaplan-Meier curves were established to assess and compare the long-term overall cumulative survivals of patients with and without CRRT at different postoperative time points. Besides the log-rank tests were used to compare the differences in the long-term overall cumulative survivals between the patients with and without CRRT, however, the Kaplan-Meier analysis should also be performed to determine the correlation of CRRT with long-term overall cumulative survivals.

## Data Availability

Not applicable.

## Abbreviations

CRRT: Continuous renal replacement treatment
CPB: Cardiopulmonary bypass
